# Current Perspectives on Reducing the β-ODAP Content and Improving Potential Agronomic Traits in Grass Pea (*Lathyrus sativus* L.)

**DOI:** 10.3389/fpls.2021.703275

**Published:** 2021-10-18

**Authors:** Arpita Das, Ashok K. Parihar, Surendra Barpete, Shiv Kumar, Sanjeev Gupta

**Affiliations:** ^1^Bidhan Chandra Krishi Viswavidyalaya, Nadia, India; ^2^ICAR-Indian Institute of Pulses Research, Kanpur, India; ^3^Food Legumes Research Platform (FLRP), International Centre for Agricultural Research in the Dry Areas (ICARDA), Sehore, India; ^4^International Centre for Agricultural Research in the Dry Areas (ICARDA), Rabat-Institutes, Rabat, Morocco

**Keywords:** grass pea, genetic improvement, genetic resources, genomic resources, neurotoxin, pre-breeding

## Abstract

Grass pea is well-established as one of the most resilient and versatile crops that can thrive under extreme climatic circumstances such as cold, heat, drought, salt-affected soils, submergence, and excessive rainfall along with resistance to several diseases and pests. However, despite the awareness of its virtues, its cultivation globally has decreased recently owing to the presence of a neurotoxin, β-N-oxalyl-L-α, β-diaminopropionic acid (β-ODAP), in the seedlings and seeds of this legume, which has been reported to cause neurolathyrism, a non-reversible neurological disorder in humans and animals. Significant repositories of *Lathyrus* germplasm are available across countries that have provided access to a wide range of agro-morphological traits as well as the low β ODAP content. Efforts have been made worldwide to use these germplasms for the genetic enhancement of grass pea to make this food safe for human consumption. Efforts on molecular breeding of this crop are also lagging. However, during the last decade, the research scenario has changed with some efforts being made toward improving this climate resilient pulse in terms of genomic resources. Molecular markers have also been used to evaluate the interspecific diversity as well as the phylogenetic relationship among the species and mapping studies. Intron-targeted amplified polymorphic, genomic simple sequence repeat, resistance genes analogs, and disease resistance markers developed for other legume species have been successfully cross-amplified in grass pea. Transcriptomic studies have recently been undertaken on grass pea by deploying several second-generation sequencing techniques. In addition, a few studies have attempted to unveil the genes and the underlying mechanism conferring biotic and abiotic stress or regulating the pathway of β-ODAP in grass pea. Proteomics has accelerated the identification studies on differential proteomes in response to salinity and low-temperature stress conditions for unveiling the common signaling pathways involved in mitigating these abiotic stresses and in discovering differentially regulated proteins. In grass pea, a metabolomics approach has been used to identify the metabolic processes associated with β-ODAP synthesis. Genome sequencing of grass pea is under way which is expected to be vital for whole-genome re-sequencing and gene annotation toward the identification of genes with novel functions. Recently, a draft genome sequence of grass pea was developed, and some efforts are underway to re-sequence a diverse panel of grass pea comprising 384 germplasm lines. Owing to the scantiness of a successful transformation protocol, research on the application of modern approaches of genome editing like the Clustered Regularly Interspaced Short Palindromic Repeats (CRISPR) or CRISPR-associated protein 9 (CRISPR/Cas9) system for the engineering of signaling pathways or regulatory mechanisms seeks immediate attention to reduce the β-ODAP content in seeds and to improve the potential agronomic traits in grass pea.

## Introduction

Grass pea (*Lathyrus sativus* L.), a nutritious legume, popularly known as Indian vetch, chickling vetch, and *khesari*, is considered as an “insurance crop” that can endure under marginal lands, rendering economic, social, and nutritional security to poorer farmers (Vaz Patto and Rubiales, 2014a; Mahapatra et al., 2020). More than 100 million people from drought-prone areas of Asia and Africa rely on grass pea as their energy source. It is a geographically successful versatile crop that can also withstand temperature extremities such as cold and heat waves, submergence, and excessive rainfall (Lambein et al., 2019). In addition, it can be sustained in saline soil and in other adverse-edapho climatic situations with nutrient deficiency or with heavy metal accretion (Ahmed et al., 2014). So, in extreme situations it can be the only available energy source for mankind. However, the genetic potential of this crop as “climate resilient” has long been neglected which has now created an urgency to increase its production potential by improving the agronomic traits for boosting up the economy of marginal land resources.

Grass pea is regarded as an ideal candidate crop of rice fallows of South East Asia where it holds immense potential by thriving well on residual soil moisture. The common practice, therefore, is to broadcast grass pea seeds into a standing rice crop immediately before harvesting of rice as a “relay crop” or “paira crop.” This ensures germination of fallow grass pea in rice fallow niches using the residual moisture and avoiding tillage operations during its cultivation (Maji et al., 2019). Under the rice fallow condition in West Bengal, India, the seed yield of grass pea was reported to be ~1,696 kg ha^−1^, with standard package of practices (Banerjee et al., 2019). The agronomic traits of this crop should thus be further improved for better adaptation in rice fallow ecologies.

Grass pea is the cheapest source of protein in the daily diets of millions of vegetarian people who cannot afford or do not prefer non-vegetarian products to access a balanced nutrition. The protein concentration in this legume is 17.7–49.3% which is higher than that of other pulses such as dry pea, faba bean, or lupine (Pastor-Cavada et al., 2011; Rizvi et al., 2016). The protein of grass pea contains 17 amino acids in sufficient amounts, especially lysine at higher levels when compared to other legumes or cereals crops (Yang and Zhang, 2005). It is a unique dietary source of the amino acid L-homoarginine (Har) which is one of the first strange non-protein amino acids (Rao et al., 1963). Har can be used as a substrate for sustained and regulated nitric oxide production and play a crucial role in treatment of cardiovascular diseases (Lambein, 2000; Rao, 2011). Besides, Har can be applied to overcome the expansion of cancer tumors, owing to the scarcity of oxygen at the tissue level (Jammulamadaka et al., 2011). Therefore, as a nutraceutical product, grass pea can be regarded as an excellent example of a potential “functional food” (Llorent-Martínez et al., 2017). Notwithstanding such virtues, its cultivation has decreased in the recent past across the world owing to the presence of a neurotoxin, β-N-oxalyl-L-α, β-diaminopropionic acid (β-ODAP) in its seedlings and seeds which has been reported to cause neurolathyrism. Therefore, it becomes pertinent to reduce β-ODAP content in seeds of this crop to ensure that the grass pea continues to provide food and nutritional security to the multitudes of low-income communities (Rizvi et al., 2016; Lambein et al., 2019).

## ODAP Content: a Major Limitation for Adoption and Utilization

Grass pea cultivation has been banned due to the association of its consumption with neurolathyrism, a non-reversible neurological disorder in humans and animals induced by β-ODAP [also known as b-N-oxalyl-amino-L-alanine (BOAA)] (Lambein and Kuo, 2009). The grass pea toxin exists in isomeric α and β forms (Bell and O'Donovan, 1966) with the toxic content of β-isomers being as high as 95% of the total ODAP (De Bruyn et al., 1994). The presence of β-ODAP in seeds as free amino acid and in high amounts in drought tolerant grass pea is believed to be responsible for this crippling disease (Lambein et al., 2019). During famine and drought years, people depend on grass pea seeds as the only source of protein and its consumtion for a prolonged period may trigger a characteristic motor system disease (a form of spastic paraparesis) (Vaz Patto and Rubiales, 2014b). β-ODAP accumulation in grass pea has been reported to be probably related to the level of total free nitrogenous compounds present in this crop. Therefore, nitrogen and phosphate may be the crucial nutrient factors that influence the neurotoxin content under field conditions. Studies have suggested that nutritional deficiencies of cysteine (Cys) and methionine (Met) may intensify the neurotoxicity level of ODAP. Until the biosynthetic pathway leading to the production of ODAP is identified it can be postulated that the ODAP biosynthesis is linked to sulfur metabolism. Unfortunately, the sulfur metabolism and its contribution to ODAP biosynthesis in grass pea are poorly understood (Xu et al., 2017). In a study, the number of amino acids (such as serine and Cys) was shown to be inversely proportional to β-ODAP accumulation, whereas, β-cyanoalanine synthase was identified as the key enzyme for ODAP accumulation in grass pea (Liu et al., 2017).

The ODAP content differs widely among the accessions depending on the genetic structure and growing environments (Dahiya and Jeswani, 1974; Ramanujam et al., 1980). Studies have reported wide variations ranging from 0.02 to 2.59% within existing germplasm (Pandey et al., 1997; Hanbury et al., 1999; Kumar et al., 2011, 2013). Both the environment and the genotypes could play an important role in the biosynthesis of ODAP. On the basis of multi-locational trials conducted in “DZARC” Ethopia 2003, it has been reported that β-ODAP level in same cultivars increases or doubles depending upon the varying growth environment under low to high stress conditions. However, drought condition, presence of excess iron or cadmium, and depletion of zinc in the soil can stimulate the increased production of β-ODAP in grass pea (Liu et al., 2017). Although the exact physiological and molecular mechanisms for the biosynthesis of ODAP content in grass pea remains unknown, studies have hinted that abiotic stresses may cause imbalance to adjust the plant's osmotic potential, which triggers ODAP biosynthesis in grass pea (Jiang et al., 2013; Piwowarczyk et al., 2014).

## Genetic Resources for Future Crop Improvement

### Crop Wild Relatives and Gene Pool

**Figure 1:**
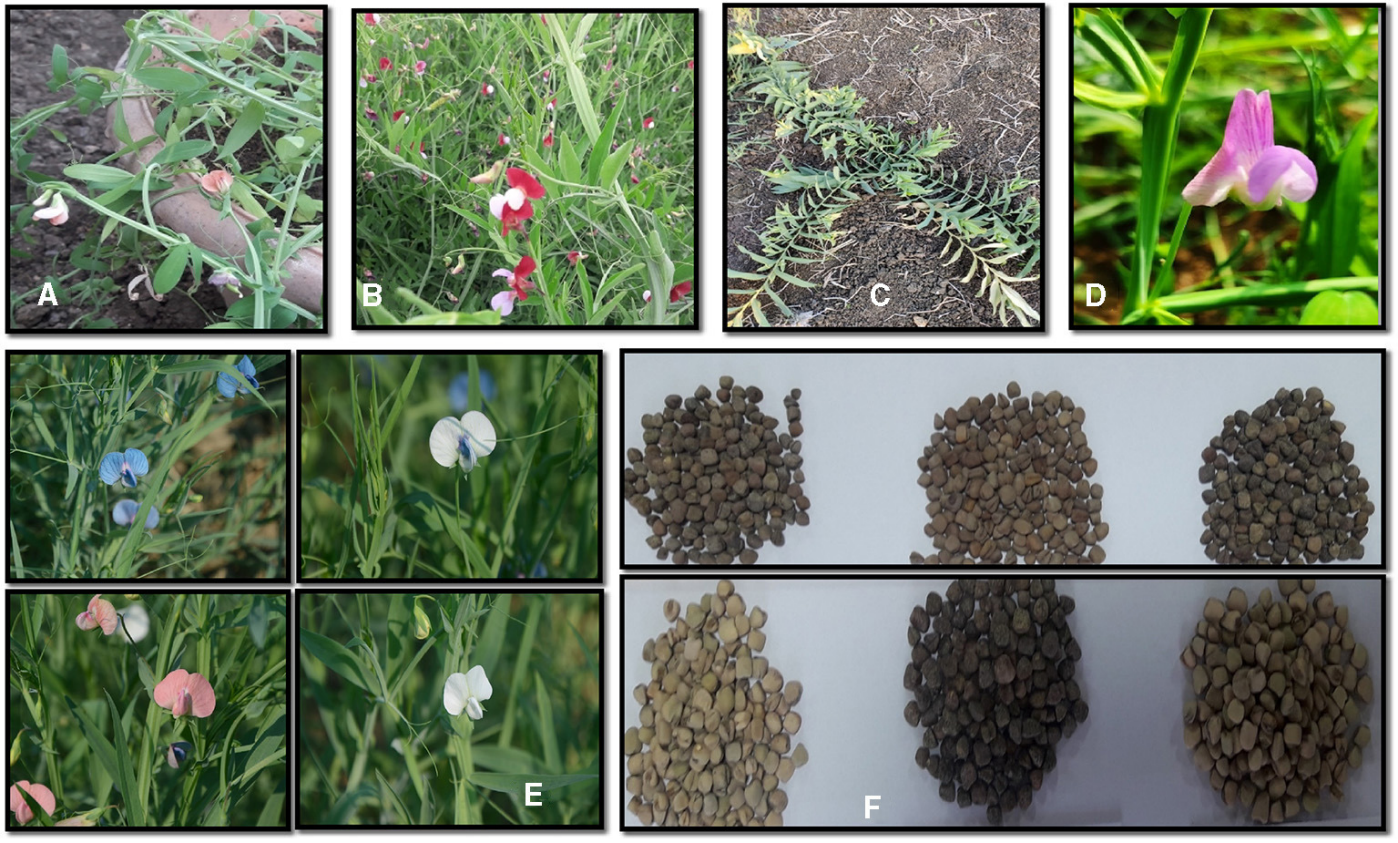
Morphological variation in Crop Wild Relatives (CWR) as well as cultivated species of *Lathyrus*. **(A)** Variation in flower color of *L. cicera*. **(B)** Variation in flower color of *L. articulatus*. **(C)** Morphological variation in *L. ochrus*. **(D)** Variation in flower color of *L. inconspicuous*. **(E)** Morphological variation in flower color among *Lathyrus sativus* L. **(F)** Variation in seed coat color and shape among *Lathyrus sativus* L.

The exploitation of crop wild relatives (CWR) of grass pea warranted further domestication and utilization of this crop as food (as low-ODAP cultivars) and fodder (as high biological yield cultivars) (Figures 1A–D). Based on taxonomical and morphological characteristics, *Lathyrus* species can be classified into 5 groups namely *Aphaca, Nissolia, Clymenum, Cicerula*, and *Lathyrus* (Asmussen and Liston, 1998; Kenicer et al., 2005). The first 4 groups belong to annual species, whereas the *Lathyrus* species are mostly perennials (Kupicha, 1983; Asmussen and Liston, 1998). The progenitor of *L. sativus* remains unknown, however, some Mediterranean species, such as, *L. cicera, L. marmoratus, L. blepharicarpus*, and *L*. *pseudocicera* qualify as candidates on the basis of their morphological resemblances with cultigens (Kumar et al., 2013).

The CWR may play an important role in the genetic improvement of cultivated species. For example, a toxin-free gene that has been identified in *L. tingitanus* that can be utilized for the development of toxin-free grass pea varieties (Zhou and Arora, 1996). However, *L. cicera* is also an excellent source because of its low ODAP content, earliness, and cold tolerance and can be utilized for grass pea improvement. Alien gene transfer is rarely attempted in grass pea, despite the successful setting of viable seeds in (interspecific hybridization) between *L. sativus, L. cicera*, and *L. amphicarpus* (Khawaja, 1988; Yunus, 1990; Addis and Narayan, 2000). From the available information on crossability (intraspecific hybridization), alteration of chromosome behavior of the hybrids and setting of viable seed, alien gene transfer is possible for crop improvement in *L. sativus* by using *L. cicera* and *L. amphicarpus* that are readily crossable species with grass pea.

### Genetic Diversity

Grass pea displays excellent morphological variation in terms of leaf length, flower color, podding structure, seed size and color (Figures 1E,F). These characteristics, as well as the yield, ODAP and protein contents, have been suggested to describe the significant variability of the *L. sativus* and *L*. *cicera* germplasms (Grela et al., 2010). A large number of grass pea genotypes have been evaluated for major agronomic traits, nutritional value, and antinutritional (ODAP) concentration (Table 1). The seed size is a mostly distinguishing feature of grass pea. Large-seeded (lakh type) forms were originated from the Mediterranean region (Syria, Turkey, Italy and Spain), medium-seeded forms were originated in northern France and Germany, and the small-seeded forms (Lakhori type) are characteristic of the South Asian and Polish cultivars (Hanbury et al., 1999). Hammer et al. (1989) indicated that the large-seeded grass pea genotypes from South Italy with a larger vegetative canopy are found around the Mediterranean region with an exceptionally high seed index. The small-seeded grass pea genotypes are highly prevalent in South Asian and South-east Asian countries (Barpete, 2015). Flowers with blue, pink, red, and white colors or various combinations of these colors are prevalent in grass pea. The blue-flowered ecotypes are found in South-east and South Asia (Polignano et al., 2005; Kumar et al., 2020); whereas, white flowered types are generally found in the Mediterranean region (Smartt, 1984). High variability of the ODAP content was recorded at both inter-specific and intra-specific levels (Sammour et al., 2007a).

**Table 1:** Genetic variation on important agronomic and seed quality traits in grass pea.

**Traits**	**Range**	**References**
Days to 50% flower	47–97 days (South Asia)	Dahiya and Jeswani, 1974; Sarwar et al., 1995
	104–129 days (Mediterranean and European type Environment)	Kumar et al., 2013, 2020; Barpete et al., 2020b
Days to maturity	86–127 (South Asia)	Pandey et al., 1995
	136–177 days (Mediterranean type environment)	Barpete et al., 2020b
Plant height (cm)	15–68 cm (South Asia and Mediterranean)	Pandey et al., 1995; Campbell, 1997; Barpete et al., 2020b
	24.5–172 cm (European type)	Campbell, 1997
100 seeds weight	2.95–8.50 (South Asia)	Pandey et al., 1995; Sarwara et al., 1995
	4.07–28.8 g (Mediterranean and European type environment)	Campbell, 1997; Barpete et al., 2020b
Pods/plant	2.4–59 (India)	Pandey et al., 1995
	20–224 (Mediterranean type environment)	Barpete et al., 2020b
Seeds/Pod	1.60–4.60 (South Asia)	Pandey et al., 1995
	1.50–4.20 (Mediterranean type environment)	Barpete et al., 2020b
Seed Yield (g/Plant)	6.2–200 g (South Asia)	Pandey et al., 1995
	3.06–50.26 g (Mediterranean type environment)	Campbell, 1997; Barpete et al., 2020b
Biological yield (g/plant)	16.18–154.69 (Mediterranean type)	Robertson et al., 1995; Barpete et al., 2020b
	0.4–51 g (South Asia)	Pandey et al., 1995; Campbell, 1997
Branches per plant	1.8–28.4 (India)	Mehra et al., 1995; Pandey et al., 1995; Campbell, 1997
	5.5–40 (Canada)	Campbell, 1997
Double flower or double pods/ single node	2 flowers/peduncle	Campbell and Briggs, 1987
β-ODAP	0.02–2.59%	Hanbury and Siddique, 2000; Arslan et al., 2017; Kumar et al., 2020
Protein	17.7–34.60%	Sammour et al., 2007a,b; Pastor-Cavada et al., 2011; Barpete et al., 2020b
Fat	2.7%	Rahman et al., 1974; Rotter et al., 1991
Calories	362.3–368.4 kcal/kg	Rahman et al., 1974; Majumdar, 2011
Carbohydrates	51–73%	Tamburino et al., 2012; Al-Snafi, 2021
Starch	35–52%	Urga et al., 2005; Girma and Korbu, 2012; Al-Snafi, 2021
Total lipid	1.6–2.0%	Tamburino et al., 2012
Fatty acids (saturated)	16–54%	Grela et al., 2010; Tamburino et al., 2012
Fatty acids (unsaturated)	45.7–66.7%	Grela et al., 2010
Iron	41–73 ppm	Urga et al., 2005; Grela et al., 2010; Sen Gupta et al., 2021
Zinc	19–54 ppm	Urga et al., 2005; Grela et al., 2010; Sen Gupta et al., 2021
Homoarginine	7.49–12.44 mg/g	Sacristán et al., 2015; Sen Gupta et al., 2021
Potassium	8.33–11.05 ppm	Grela et al., 2010, 2012
Magnesium	0.86–1.61 ppm	Hanbury and Siddique, 2000; Grela et al., 2012
Manganese	7.86–42.5 ppm	Hanbury and Siddique, 2000; Grela et al., 2010
Tannin	2.72–5.62 g/kg	Deshpande and Campbell, 1992; Grela et al., 2010; Al-Snafi, 2021
β-carotene	240.8–410.1 μg/kg	Arslan, 2017

Genetic diversity of grass pea has been evaluated and documented on the basis of morphological markers, several biochemical and molecular marker loci encoding storage proteins, isozymes or DNA based markers (Sammour et al., 2007b). The induced dwarf mutants of grass pea, with allozyme variants regarding leaf esterase and root peroxidase isozymes, can be effectively utilized for discriminating dwarf mutants from one another (Talukdar, 2010). Three isozymes namely esterase, aspartate aminotransferase, and acid phosphatase used for measuring variability among the Ethiopian grass pea resulted in non-significant correlation with morphological diversity (Tadesse and Bekele, 2001). In grass pea breeding programs, diverse molecular markers like random amplified polymorphic DNA (RAPD); restriction fragment length polymorphism (RFLP); amplified fragment length polymorphism (AFLP); inter simple sequence repeat (ISSR); expressed sequence tag-simple sequence repeats (EST-SSR); sequence-related amplification polymorphism (SRAP); and sequence tagged site (STS) have been utilized for deciphering diversity and phylogenetic relationship among the species (Hanada and Hirai, 2000; Chtourou-Ghorbel et al., 2001; Tavoletti and Iommarini, 2007; Lioi et al., 2011; Shiferaw et al., 2012; Soren et al., 2015; Marghali et al., 2016; Gupta et al., 2018).

### Potential Donors for Further Utilization

Despite several known advantages, only a few scientific approaches have been used for improving grass pea to date. Therefore, the conventional and modern breeding programmes on *Lathyrus* should focus on developing germplasm/cultivars with a low β-ODAP content and a higher grain yield. Presently, several grass pea low-ODAP accessions (0.04–0.1%) are available throughout the world (Table 2) including in India, Bangladesh, Nepal, Ethiopia, Australia, Canada, Poland, and Turkey. Furthermore, some wild relatives namely *L*. *cicera, L*. *amphicarpus* and *L*. *ochrus* species have zero or low-ODAP (≤ 0.01%) genes that can be utilized for the development of toxin free *Lathyrus* varieties (Campbell, 1997; Kumar et al., 2013). Although grass pea is a self-pollinating species, a significant proportion of outcrossing (2.0–27.8%) by bees has been reported in this crop (Chowdhury and Slinkard, 1997; Hanbury et al., 1999). In addition, similar outcrossing rates in *L. cicera* have been reported owing to its similarities with *L*. *sativus*in in terms of floral biology (Hanbury et al., 1999). Moreover, most of the conventional breeding programmes of grass pea have focused on increasing the yield by using the selection criterion (number of branches per plant). However, some of the available *Lathyrus* germplasms have attractive yield traits such as single node double flowers or pods (L900239 and L920278) and >30 g/100-seed weight (LS-2026, LS-8, LS-97 and Quila-blanco). These traits can be further utilized in grass pea improvement programs (Campbell and Briggs, 1987; Ulloa and Mera, 2010).

**Table 2:** Potential source for grass pea improvement.

**Trait**	**Germplasm/variety/wild relatives**	**Country**	**Reference**
Low ODAP (0.04–0.1%)	BARI Khesari-1, BARI Khesari-2, BARI Khesari-3, BARI Khesari-4, BARI Khesari-5 BINA Khesari-1	Bangladesh	Malek et al., 1996; Kumar et al., 2013
	Ratan, Prateek, P-24, Mahateora, Nirmal, Bidhan Khesari-1	India	Lal et al., 1985; Pandey et al., 1996; Indian Council of Agricultural Research, 2009
	Wasie	Ethiopia	Tadesse and Bekele, 2003
	CLIMA pink, 19A, 20B	Nepal	Yadav, 1996
	Ceora, Chalus (*L. cicera*)	Australia	Hanbury and Siddique, 2000
	LS 8246 and AC-Greenfix	Canada	Campbell and Briggs, 1987
	Derek and Krab 9	Poland	Kumar et al., 2013
	Gurbuz-1	Turkey	ICARDA, 2007
	*L. cicera, L. amphicarpus L. ochrus*	ICARDA	Campbell, 1997; Kumar et al., 2013
Double flower or podding in single node	L900239 and L920278	Canada	Campbell and Briggs, 1987
	IFLA-1864, IFLA-143	India	Barpete, 2020 unpublished report
Seed weight (≥ 30 g/100 seed)	LS-2026, LS-8, LS-97, Quila-blanco	Chile	Ulloa and Mera, 2010
Earliness (≤ 100 days)	BANG-267, BANG-310, IFLA-2475	India	Barpete, 2020 unpublished report
**Insect and pest resistance Lathyrus germplasm/wild relatives**			
Thrips (*Caliothrips indicus)* resistance (insect)	RLK-1, RLK-281, RLK-617, RPL-26, RLK-273-1, RLK-273-3, JRL-6 and JRL-41	India	Lal et al., 1985; Pandey et al., 1997; Banerjee et al., 2019
Bruchid resistance (Insect)	cv. Rodos (from *L. cicera*)	Greece	Tsialtas et al., 2020
Rust (*Uromyces pisi*)	BG-15744 and BG-23505 (Partially resistance)	Spain	Vaz Patto and Rubiales, 2009, 2014a
Powdery mildew (*Erysiphe pisi*)	IPLy2-10, RPLK-26, RL-41, RLS-2, RPLK-26 and RL-21, LS8246, landrace a-60	India	Narsinghani and Kumar, 1979; Lal et al., 1985; Sastri, 2008
Downy mildew (*Peronospora lathyri-palustris*)	RLS-1, RLS-2, JRS-115, JRL-43, and JRL-16	India	Lal et al., 1985; Asthana and Dixit, 1997
Ascochyta blight (*Mycosphaerella pinodes*)	ATC 80878	Australia	Gurung et al., 2002; Skiba et al., 2004a
Cyst nematode (*Heterodera ciceri*)	IFLA 347 (partially resistant)	ICARDA	Vito et al., 2001
Root knot nematode (*Meloidogyne artiella*)	(PI 236481 & UT2921 from *L. latifolius*), (PI 358879 from *L. sylvestris*) (PI 440462 from *L. hirsutus*) species	Washington, USA	Rumbaugh and Griffin, 1992
Crenata broomrape (*Orobanche crenata*)	*L. clymenum and L. ochrus* (resistance species)	Spain	Linke et al., 1993; Sillero et al., 2005

## Breeding Approaches for Cultivar Development

Notwithstanding the numerous known advantages of grass pea, little effort has been made toward its improvement owing to the stigma associated with ODAP (Vaz Patto et al., 2006). However, most of the initial progress in the field of grass pea research toward the development of low ODAP varieties was made through direct selection from landraces and lines (Vaz Patto et al., 2006). Conventional breeding centralized fundamentally on the hybridization of selected genotypes followed by screening and evaluation of the subsequent progenies for the traits of interest. In case of breeding targeted toward reducing ODAP content, crossing of low ODAP accessions with high-yielding material demonstrated good agronomic potential (Campbell, 1997). The high yield potential has been a selection criterion for most crop improvement programmes. On the other hand, some of the components that influence yield such as double podding or increased seeds per pod have rarely been utilized. The biomass yield of *L. sativus* has also received attention due to the significant potential of this crop as forage and straw in the North African and South Asian regions (Campbell, 1997; Vaz Patto et al., 2006). Efforts were concentrated on the development of high yielding varieties with a low β-ODAP content. The Indian landmark variety of grass pea, “Pusa 24,” was selected from a field in 1966 and acknowledged as the first cultivar to possess low ODAP content in its seeds (0.2%). Notably, the Pusa 24 variety serves as the base parent of several other low-ODAP varieties in India and other countries. Subsequent research efforts have led to the development of varieties suitable for upland (LSD1, LSD2) and rice fallow (LSD3, LSD6, Pusa-305, and Selection 1276) with low (up to 0.2%) ODAP content (Gautam et al., 1998). In Chile, the cultivar “Quila-blanco” was developed in 1983 through selection from the locally grown heterogeneous population. The major characteristics of this cultivar are synchronous maturity and bold white seeds (100 seeds weight, 28.7 g) with a protein concentration up to 24.0% (Campbell et al., 1993). Meanwhile, various attempts have also been made to establish an association between easily observable characteristics and ODAP for the ease of selection, although it remains unsuccessful owing to the polygenic inheritance of ODAP, which is highly influenced by the genotype and environment and their interactions (Hanbury et al., 1999). The conventional breeding programmes of grass pea were well-established across several countries, including Australia (McCutchan, 2003), Bangladesh (Rahman et al., 2017), Canada (Campbell and Briggs, 1987), China (Yang and Zhang, 2005), Ethiopia (Tadesse and Bekele, 2003), India (Pandey et al., 1996; Santha and Mehta, 2001), Nepal (Yadav, 1996), Syria (Abd El Moneim et al., 2001; Kumar et al., 2011), Poland (Grela et al., 2010), Italy (Granati et al., 2003), USA (Krause and Krause, 2003), and Chile (Mera et al., 2003). Some of these breeding programmes are still active, however, they are meager in comparison with that for other legume crops (Vaz Patto et al., 2006). Several varieties and lines have been developed by combining low β-ODAP (<0.1%) with high-yield potential (up to 1.5 tons/ha) and resistance to an array of biotic and abiotic stresses (Kumar et al., 2013; Dixit et al., 2016; Sen Gupta et al., 2021).

### Pre-breeding and Distant Hybridization

The substantial genetic diversity available within *L. sativus* is being utilized for the improvement of this crop through the exploitation of the primary genepool by using conventional approaches (Chowdhury and Slinkard, 2000). However, to broaden the genetic base of the crop the introgression of desirable alleles from outside the primary gene pool through pre-breeding and distant hybridization is warranted. Successful inter-specific crosses have been established between grass pea and other *Lathyrus* spp., particularly *L. pseudocicera*. Embryo rescue has increased the range of species in successful inter-specific crosses (Addis and Narayan, 2000). The results of inter-specific hybridization in grass pea suggest that the identification and transfer of desirable traits from exotic and wild germplasm offer numerous opportunities for *Lathyrus* improvement, particularly for crossable species such as *L. cicera* and *L. amphicarpus* (Yunus, 1990; Yunus and Jackson, 1991). Crosses in *Lathyrus* have also been performed using other species such as *L. chrysanthus, L. gorgoni, L. marmoratus*, and *L. pseudocicera* (Heywood et al., 2007); however, only ovules were produced in these experiments. The appraisal of wild *Lathyrus* for ODAP content has clearly revealed the lowest ODAP amount in *L. cicera*, followed by that in *L. sativus* and *L. ochrus* (Hanbury et al., 1999).

### Mutation Breeding

A wide range of morphological mutations has been noticed that affects growth habit, maturity, branching, stem shape, leaf size, stipule shape, flower color and structure, pod size, seed size, and seed color (Waghmare et al., 2001; Talukdar, 2009). Corresponding to the morphological changes, chromosomal changes including translocations were induced in grass pea through mutagenesis (Biswas and Biswas, 1997; Talukdar, 2009). Mutation breeding with EMS (0.01%) and gamma rays (250 Gy) was performed to develop two varieties such as “Poltavskaya” and “Bina Khesari-1” in Russia and Bangladesh, respectively (Kumar et al., 2011, 2013). Asnake (2012) reported putative mutants with improved Met content compared with the parent wherein the Met supply capacity of grass pea shifted from 25% in the parent line to 50% in the altered putative mutant lines.

### Marker-Assisted Breeding

The efficiency of Marker-assisted selection (MAS) depends on closely linked molecular markers with the trait of interest remaining quintessential. Generally, the EST-SSR marker system showcases a high degree of conservation and can be transferred among species, although the numbers of ESTs for *L. sativus* (178) and *L. cicera* (126) remain extremely limited when compared with those available for *L. odoratus* (8702) (Lambein et al., 2019). With the development of high-throughput and dense genotyping systems; association mapping has gained an advantage over the bi-parental population through generations of a large number of recombinants in a short period (Morrell et al., 2012; Cobb et al., 2013). Thus, the development of a comprehensive genetic map for *Lathyrus*, with the identification of valuable genes and quantitative trait loci (QTLs) for MAS and with the possible alignment with other species, is urgently required. Linkage maps, gene cloning, and MAS are expected to hasten the introgression of novel genes for low ODAP and increased Met contents and, therefore, they can be used to improve the quality of locally adapted cultivars.

### Improvement *via in vitro* and Transgenic Technology

*In vitro* tissue culture techniques have excellent potential to enhance and improve the agronomical traits through creation of somaclonal variation, screening for salt and drought tolerant genotypes, identification of micronutrient concentrations/toxicity, generation advancement or regeneration of true to the type plant in grass pea (Barpete et al., 2016, 2020a, 2021). However, grass pea is highly recalcitrant and difficult to regenerate under *in vitro* conditions (Barpete et al., 2016). The first fertile plant regeneration protocol from meristematic tissues was developed and optimized in grass pea by Zambre et al. (2002). Thereafter, different explants from axenic seedlings (including leaf, internode, cotyledon, hypocotyl, and epicotyl) were utilized for plant regeneration. Among the different explants of grass pea, the epicotyl was reported to be the most responsive with high shoot proliferation frequency. Development of somaclones in *L. sativus* is limited (Santha and Mehta, 2001; Tripathy et al., 2016; Barpete et al., 2020a), but in India, such efforts have developed somaclones derived low-ODAP variety (Ratan) (Mehta et al., 1994; Santha and Mehta, 2001). Tripathy et al. (2016) have also developed a series of somaclones for morphological, cytological (genetic) variation, and biochemical levels in four grass pea (Nirmal, P 24, Nayagarh local, and Dhenkanal local) genotypes. A high-yielding and low ODAP somaclone (cv. NGOG 5) was developed that may be the potential candidate for future grass pea breeding programs (Tripathy et al., 2016). Ochatt et al. (2002) and Barpete et al. (2020a) developed an *in vitro* system for shortening the generation cycles and hastening the breeding process coupled with the *in vitro* stages that provide up to 4–5 cycles per year of grass pea. Although this biotechnological approach is suitable only when a small number of seeds/plants is required, it can positively contribute toward accelerating the breeding programme of ODAP-free grass pea varieties. Mostly, the regeneration protocols for *L*. *sativus* are genotype dependent; therefore, development of genotype-independent or ubiquitous protocols suitable for grass pea regeneration are necessary (Ochatt et al., 2013; Barpete et al., 2016).

In legumes, genetic transformation frequency is low due to the non-responsive nature for organogenesis or somatic embryogenesis. The genetic transformation protocol was standardized using the epicotyl explant of Indian grass pea accession, co-cultured with two disarmed *Agrobacterium tumefaciens* strains (EHA 105 and LBA 4404) (Barik et al., 2005). After several attempts by Ethiopian researchers, a prolific grass pea regeneration protocol was standardized for transient genetic transformation of two grass pea varieties of Ethiopian origin (Girma and Korbu, 2012). The Ethiopian scientists attempted to enhance the quality of grass pea seed protein, and accordingly the genetic transformation was planned for improving the Met content through gene coding (Girma and Korbu, 2012). Recently, grass pea *Agrobacterium*-mediated genetic transformation was shown to improve the nutritional quality and tolerance to fungal pathogens, without any adverse effect on the seed protein quality, whereas, reduced β-ODAP level (up to 73%) was additionally reported by Kumar et al. (2016). Besides, antinutritional metabolite, oxalic acid (OA) is a known precursor of β-ODAP. The reduced level of OA in transgenic seeds of grass pea (up to 75%) was interrelated to an increase in seed micronutrients, such as calcium, iron, zinc, manganese, and magnesium (Kumar et al., 2016). Hence, there is huge possibility of genetic transformation for nutritional improvement and safe utilization of grass pea.

## OMICS Enabled Improvement in Grass Pea

Molecular breeding efforts for the grass pea crop continue to lag, and this crop has long been neglected concerning biotechnological investments and is therefore considered an orphan crop. Large genome size (1C = 8.12 Gbp) coupled with the limited characterization of the available germplasm and low investment due to its confined cultivation in resource-poor areas along with the meager numbers of scientific communities associated with grass pea improvement have restrained the development and application of new molecular tools and techniques toward the improvement of this legume (Sarkar et al., 2019). Genomic resources for grass pea remain scarce (in May 2020 the National Center of Biotechnology Information (NCBI) database contained only 178 EST sequences of grass pea) and the marker density is poor, which restricted development of a densely saturated linkage map and thus limit the accuracy and efficiency of quantitative trait loci (QTL) mapping (Skiba et al., 2004a,b; Vaz Patto et al., 2006). Further, the grass pea reference genome sequence is not available (Hao et al., 2017). In following sections, we present an in-depth summary of the latest developments in genomics and molecular breeding as well as the challenges and scopes toward the application of new tools concerning the genetic improvement of this crop.

### Molecular Marker Development

In grass pea, the numbers of molecular markers are surprisingly low, which necessitates the development of a larger number of functionally relevant molecular markers toward the successful deployment for molecular breeding strategies (Lioi and Galasso, 2013). To overcome the bottleneck of insufficiency of anchor markers, effort have been made to harness EST-SSR markers from closely related legume species for diversity estimation and evolutionary and mapping studies. The availability of cross-species amplified markers along with computational search of the nucleotide sequence database of ESTs available at NCBI GenBank for grass pea (http://www.ncbi.nlm.nih.gov/genbank/dbest/dbest_summary/) has enabled the faster and more cost-effective development of genic SSRs (EST-SSRs) in this genetically underexploited crop (Skiba et al., 2003). Moreover, CAPS and derived-CAPS (dCAPS) were also designed by sequencing the monomorphic SSR fragments and examining the RIL population of *L. cicera* (Almeida et al., 2014a,b; Shiferaw et al., 2017).

In the absence of a reference genome in grass pea, the breakthrough of next-generation DNA sequencing (NGS) technologies has led to the development of rapid genome-wide SSRs and SNPs detection which is expected to facilitate positional cloning and QTL mapping (Xu et al., 2018). Transcriptomic studies in grass pea have recently been undertaken (Almeida et al., 2014b, 2015; Yang et al., 2014; Chapman, 2015; Hao et al., 2017; Tan et al., 2017; Xu et al., 2018; Rathi et al., 2019). The first transcriptome study of grass pea with 454 FLX Titanium Pyrosequencing Technique empowered the identification of 651,827 SSRs, and subsequently, 50,144 non-redundant primer pairs were designed which finally enabled the detection of 74 polymorphic and 70 monomorphic products as well as 144 products with no polymerase chain reaction (PCR) (Yang et al., 2014).

### Genetic Mapping Strategy in Grass Pea

In the absence of a sufficient number of robust marker systems for grass pea, the numbers of densely saturated linkage map have become limited for this crop in compared with those for other well-studied legumes (Table 3). The availability of a densely saturated genome linkage map is expected to detect QTLs controlling the polygenes throughout the genome and subsequently tagging the QTL region with molecular markers for future MAS or map-based cloning of the underlying genes toward expediting the breeding programs. The first linkage map for grass pea with molecular markers was constructed by Chowdhury and Slinkard (1999). One limitation of this linkage map was that ~12% of the markers utilized in this study exhibited segregation distortion which increased the rate of false linkages in F_2_ populations and subsequently lead to the unreliable estimation of map distances. For studying the genetics of tendril trait, bulked segregant analysis was performed on the F_2_ population of sweet pea (*L. odoratus*) derived from a cross between parents with tendril (“Grace”) and without tendril (“Snoopea purple”) followed by detection of the RAPD marker (WB32a) linked with the tendril gene (Hanada and Hirai, 2003). In 2004, another group of researchers constructed a genetic linkage map from a population of 92 backcrossed individuals derived from a cross between an accession “ATC 80878” resistant to ascochyta blight (*M. pinodes*) and a susceptible accession “ATC 80407” (Skiba et al., 2004a). Two QTLs associated with ascochyta blight resistance of stem of grass pea were identified (Manly et al., 2001). RNA sequencing-derived marker (SSRs, SNPs) systems were deployed for the construction of linkage map of *L*. *cicera* (Santos et al., 2018). Comprehensive research is thus needed to map agronomically important as well as climate- resilient traits to further facilitate mapping and tagging of molecular markers linked to genes conferring these traits for achieving success in MAS for accelerating the breeding process of grass pea.

**Table 3:** Summary of molecular mapping studies carried out in grass pea.

**S No**.	**Population**	**Trait**	**Markers**	**Linkage group**	**Average marker density (cM)**	**Mapped loci**	**No. of QTLs**	**Reference**
1	Four F_2_ populations derived from eight sub accessions (PI 283564c.3 × PI 426885.2, PI 358601.5 × PI 173714.5, PI 426891.1 × PI 172930.4, and PI 283549a.6 × PI 202803a.3)		11 isozymes (ACO-1, ACO-2, AAT-1, AAT-2, EST-3, EST6, FDH, LAP-1, PGD-2, SKDH, and TPI-1)	2	**-**	11	**-**	Chowdhury and Slinkard, 2000
2	F2 progenies from seven accessions of cultivated grass pea	-	9 isozymes	3	-	6	-	Gutiérrez et al., 2001
3	Eleven parents among which four were diploid (2*n* = 14) and rest seven were primary trisomic (2*n*+1 = 15) types and M4 mutant populations	Flavonoid deficiency	Isozymes of enzyme aconitase (ACO) and S-nitrosoglutathione reductase (GSNOR)	-	-	4	-	Talukdar, 2012
4	F_2_ population (Blue flowered: PI42689.1.1.3 × white flowered PI 283564c.3.2)	Flower color	71 RAPDs, 3 isozymes and 1 morphological marker	14	898cM	69	-	Chowdhury and Slinkard, 1999
5	Sweet pea (*Lathyrus odoratus*) F2 derived from cross between (Grace × Snoopea BSA) in sweet pea (*Lathyrus odoratus*) F2 population (Grace × Snoopea purple)	Tendril	302 RAPD markers	-	-	2	-	Hanada and Hirai, 2003
6	Backcross population (ATC 80878 × ATC 80407)	Aschochyta blight resistance	47 RAPD, 7 EST-SSR, 13 STS/ CAPS	9	803.1 cM	64	QTL1 (12%) QTL2 (9%)	Skiba et al., 2004a
7	F_5_RIL (BGE008277 × BGE023542)	Rust (*Uromyces pisi*) resistance	189 SNP, 113 EST-SSR, and 5 Intron Targeted Amplified Polymorphism (ITAP) markers	9	724.2 cM	307	-	Santos et al., 2018

### Functional Genomics Appliance for Grass Pea Improvement

Most of the transcriptome studies have been conducted to generate a large number of genome-wide SSRs and SNP markers for their further utilization in molecular mapping and map-based cloning studies (Yang et al., 2014; Hao et al., 2017). Limited studies have attempted to unveil the genes and the underlying mechanism conferring biotic and abiotic stresses or the pathway of regulating β-ODAP in grass pea (Emmrich, 2017; Xu et al., 2018; Rathi et al., 2019) (Table 4). In 2014, the first study on the global expression profiling of genes in grass pea-pathogen interaction for rust resistance was conducted (Almeida et al., 2014b). Another attempt was made to achieve a molecular overview of grass pea in response to aschochyta blight (*A*. *lathyri*) infestation (Almeida et al., 2015). In addition, transcriptome studies have been conducted to detect genes and regulatory pathways controlling β-ODAP flux in different growth stages of grass pea cultivar “LZ” (Xu et al., 2018). Transcriptomic orchestral concerning the dehydration tolerance mechanism in grass pea was presented by Rathi et al. (2019). In their study, 137 transcription factors (TFs) related to dehydration response and ABA and cytokinin synthesizing enzymes were detected in relation to drought tolerance mechanism of grass pea.

**Table 4:** Brief compilation regarding the application of NGS techniques in grass pea.

**S No**.	**Genotype used**	**Trait**	**Sequencing platform**	**Contigs**	**Transcripts**	**Unigenes**	**No. of primers detected**	**No. of genes annotated**	**References**
1	Eight grass pea (*L. sativus*) accessions consisted of two Chinese, two Asian, one African and three European accessions	-	Roche 454 GS FLX Titanium platform	370,079 (453 bp)	-	-	651,827	**-**	Yang et al., 2014
2	BGE015746 and BGE024709	Rust resistance	Illumina Hiseq 2000	134,914	-		2,634 SNPs 200 EST-SSR	50,937 (60.4% into functional categories)	Almeida et al., 2014b
3	BGE015746	Aschochyta blight resistance	Illumina Genome Analyser IIx	-	-	14,386	-	13,773	Almeida et al., 2015
4	Rewa-2	-	Illumina HiSeq 2500	15,779,854	49,280	33,042	1139 SSRs	-	Chapman, 2015
5	RQ23 and RQ36	-	Illumina NextSeq 500	1,970,104	142,053	27,431	3,204 EST-SSR, 146,406 SNP	-	Hao et al., 2017
6	LZ	β-ODAP	Illumina HiSeq 3000	287,695	213,258	213,258	-	27,032	Xu et al., 2018
7	LP-24	Drought	Illumina HiSeq 2500	-	165,910	41,661	8079 SSRs	31,368	Rathi et al., 2019

A comparative proteomics study was conducted to identify the differential proteome of grass pea cultivar “LP-24” in response to salinity and low-temperature stress for unveiling the common signaling pathways to mitigate these abiotic stresses and discover 67 differentially regulated proteins (Chattopadhyay et al., 2011). In grass pea, metabolomics approach was used to identify the metabolic processes associated with β-ODAP synthesis (Liu et al., 2017).

Genome sequencing of a European grass pea cultivar (“LS007”) has been completed with a draft genome assembly of ~6.3 Gbp coupled with N50 of about 59.7 kbp and an estimated grass pea genome size of 5.456–8.471 Gbp (Emmrich et al., 2020, unpublished). These data are vital for whole genome re-sequencing and gene annotation to identify genes with novel function. Integration of omics resources facilitates the retrieval of complete information about grass pea candidate genes and the underlying intrinsic pathways that are pertinent to improve the agronomic traits and resistance mechanism. Attention should be given to comprehend the ongoing research on making a large number of genome wide high density markers accessible for harnessing genome-wide approaches, such as genome wide association studies (GWAS) and genomic selection (GS) for exploring rare allelic variation for their introgression in the cultivated species by opting modern approaches such as the AB-QTL strategy, chromosome segment substitution lines (CSSL) or exotic libraries for reducing genetic noise, as well as the rapid generation turn over (RGT) toward improving genetic gain. Targeting Induced Local Lesions in Genomes (TILLING) and Eco-TILLING are the important reverse genetic approaches, which are deemed suitable for crops such as grass pea that lacks sequence information for uncovering gene functions, unfortunately, the research is still a “proof of concept” for grass pea due to a small size of the presently available mutant populations (Gurung and Pang, 2011). Presently, the John Innes Centre has created EMS mutagenized populations in two grass pea varieties for screening of low-ODAP mutants by a new high-throughput method (Emmrich, 2017). The TILLING platform *RevGen*UK (http://revgenuk.jic.ac.uk/), which was established initially for model legumes, has now been extended to grass pea (Robson, 2014) for applying NGS based deep sequencing technique for the detection of rare mutants. This review intends to call attention toward the international collaboration for sharing the genotyping data of the core set of grass pea and for bridging the phenotyping data in different environments to render a faster breeding strategy in this underexploited legume for the development of new-generation grass pea.

## Genome Editing

Genome editing allows precise targeted changes in the genome of a plant, involving engineered nuclease and cellular machineries for DNA repair, enabling targeted changes in the DNA base sequences through substitution as well as addition of bases (Meng et al., 2017; Ji et al., 2019). In recent decades, genome editing has been applied in model plants, crops plants, and fruits (Kim et al., 2017; Lin et al., 2020; Tripathi et al., 2020). The CRISPR/Cas9 toolbox can be explored for creating desirable changes in the genome for the broadening of the gene pool as well as development of new varieties within a short breeding cycle. Recently, CRISPR/Cas9 has been explored in legumes for modifying several agronomic and resistance traits like delayed flowering (Cai et al., 2020), altered node number (Bao et al., 2019) and resistance to soybean mosaic virus (Zhang et al., 2020) in soybean; intrusion of biological nitrogen fixation in cowpea (Ji et al., 2019), and resistance against drought in chickpea (Badhan et al., 2021). Attempts have also been made to modify several seed related traits associated with nutritional as well as antinutritional factors with standard transformation protocol (Li et al., 2019; Wang et al., 2020). CRISPR/Cas9 has been successfully employed to increase the concentration of the sulfur containing amino acids like Met and cysteine (Warsame et al., 2018). Changes in the MET concentration can reduce the ß-ODAP concentration in grass pea. Although, grass pea is known to have resistance toward uptake as well as integration of foreign DNA and recalcitrant for regeneration which ultimately hinder the successful application of this cutting-edge tool in grass pea. However, scientists are attempting to expand CRISPR/Cas9 system in grass pea improvement programme for the engineering of signaling pathways or regulatory mechanisms involved in the ODAP biosynthesis as well as biotic and abiotic stresses (Emmrich, 2017).

## Way Forward

**Figure 2:**
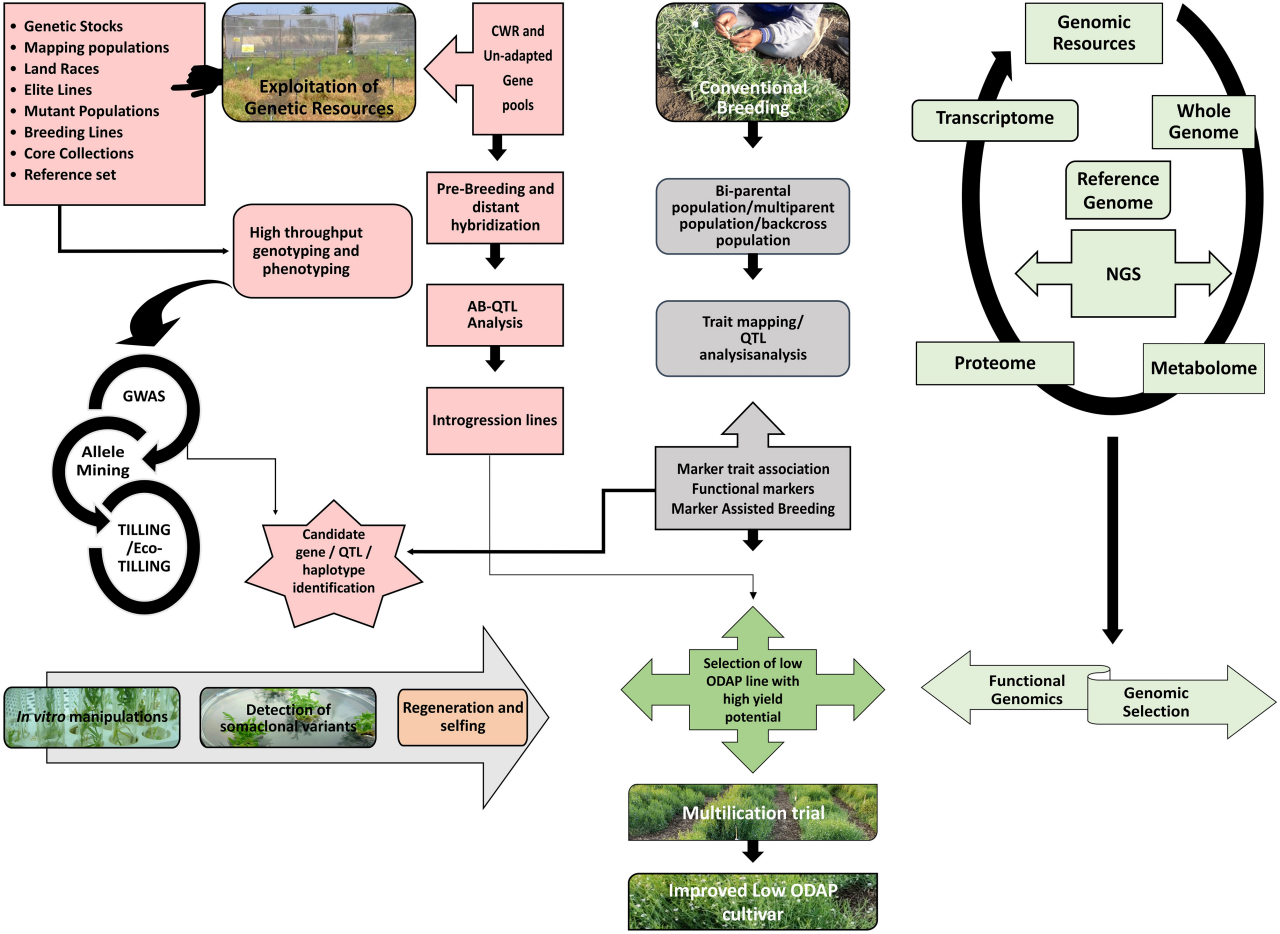
Integrative breeding and genomic approaches for development of low-ODAP grass pea cultivars. The figure depicts that exploitation of genetic resources can be achieved either through utilizing members of GP-I as well as CWR or unadapted gene pools through opting pre-breeding and distant hybridization strategy which will further lead to the discovery of candidate genes/ haplotype or QTLs. Simultaneously, conventional breeding strategy can be opted for development of different kinds of mapping populations for establishing marker-trait associations. Genomic resources can be deployed via NGS approach. Functional genomics and Genomic selection can also be utilized for unraveling the genetic mechanism of key biosynthetic pathways as well as estimation of breeding values for selection of superior cultivars. Different *in vitro* strategies can also be successful for detection of low-ODAP somaclonal variants. All these approaches ultimately lead to the development of stable low-ODAP cultivars with good agronomic base. CWR, Crop Wild Relatives; AB-QTL, Advanced Backcross QTL; GWAS, Genome Wide Association Study; TILLING, Targeting Induced Local Lesions in Genomes; QTL, Quantitative Trait Loci; NGS, Next Generation Sequencing.

Grass pea is important to the rural and poor inhabitants of several parts of South Asia and Sub-Saharan Africa and also a valuable crop in Central and West Asia, North Africa, Southern Europe and South America. It belongs to the 7 important protein sources for several of the South Asian and Sub-Saharan African countries, which makes this crop valuable for areas where the cultivation of other legumes is either risky owing to diseases, poor soil conditions, and soil problems such as water logging or difficulty owing to the threat of drought. People from these areas have requested the government, research institutions, and extension workers to focus on exploring possibilities of increasing the production of grass pea. Despite these requests, the global area under its cultivation has decreased because of the ban on its cultivation in several countries due to its association with neurolathyrism. By virtue of the negative stigma associated with grass pea, it has not received appreciable research attention, especially in the domain of genetic improvement for increasing its production potential. Therefore, grass pea programmes should focus on developing germplasm/cultivars with low β-ODAP content along with higher grain yield, biomass, earliness, disease resistance, protein content, and digestibility. A large number of grass pea genotypes were evaluated for major agronomic traits such as 100 seed weight (>30 g), pod numbers, earliness, high biomass and low ODAP content. Presently, several low-ODAP accessions (0.04–0.1%) in cultivated grass pea are available worldwide. Further improvement in cultivated species can be made with the exploitation of wild relatives namely *L*. *cicera, L*. *amphicarpus*, and *L*. *ochrus* species, which have zero or low-ODAP ( ≤ 0.01%) gene for the development of toxin free *Lathyrus* varieties. A combination of potential donors possessing other desirable agronomic traits available in the gene pool should be exploited through systematic breeding programme to improve genotype as well as development of lines for mapping of various traits. The schematic diagram explains how different approaches can be combined to establish the basis of a strategic breeding programme for grass pea (Figure 2). This figure also illustrates the use of CWR in the grass pea improvement programme. *In vitro* methods such as identification of somaclonal variants have been successfully employed in development of first low-ODAP line, Bio L 212, in India. Similar efforts require intensification in the improvement program. Moreover, Comparative genomics should be applied for the elucidation of the genetics of resistance and important agronomical characteristics to pave the way for the identification of valuable genes/QTLs. A more comprehensive genetic map with identified valuable genes and QTLs is thus required for deployment of MAS in breeding strategies of grass pea. Linkage maps, gene cloning, and MAS are expected to hasten the introgression of novel genes for low ODAP and increased Met, which can improve the locally adapted cultivars. Efforts at the international level are believed to help to decipher the genome for facilitating the identification of genes of agronomic importance. Renewed research efforts are warranted for employing next-generation genomics and phenomics in these improvement programs. Further, transcriptomics and proteomics studies are required to validate the sequencing results at the functional level. Mutation breeding and screening of mutants with the conventional and genomic tools such as TILLING and Eco-TILLING can be resorted for developing zero or low-ODAP lines in grass pea. Recently, genome editing has been reported as an efficient tool for the *de novo* domestication of many food legumes (Cai et al., 2020; Zhang et al., 2020). Similar efforts are believed to help toward silencing the genes associated with ODAP biosynthesis in grass pea. Thus, large research investment, greater cooperation among stakeholders and creation of national, regional and international synergies are required to turn this orphan crop into a mainstream pulse legume of the world.

## Author Contributions

All authors listed have made a substantial, direct and intellectual contribution to the work, and approved it for publication.

## Funding

This work has funding support from the International Center for Agricultural Research in the Dry Areas (ICARDA), Rabat Office, Rabat-Institute for open access publication fees.

## Conflict of Interest

The authors declare that the research was conducted in the absence of any commercial or financial relationships that could be construed as a potential conflict of interest.

## Publisher's Note

All claims expressed in this article are solely those of the authors and do not necessarily represent those of their affiliated organizations, or those of the publisher, the editors and the reviewers. Any product that may be evaluated in this article, or claim that may be made by its manufacturer, is not guaranteed or endorsed by the publisher.
